# Neil Patrick Eskell

**Published:** 1938

**Authors:** 


### NEIL PATRICK ESKELL, M.A.CANTAB, M.R.C.S., L.R.C.P.

ON 9th November, 1938, the death occurred of Dr. N. P. Eskell, of 167 Wells Road, Bristol. Dr. Eskell was the son of Dr. Patrick Eskell, of Bath. He was educated at Clifton College and Pembroke College, Cambridge. After leaving

Cambridge he completed the clinical part of his medical curriculum in Bristol, and obtained the qualification M.R.C.S., L.R.C.P. in 1935. He had held the posts of House Physician and Deputy Resident Surgical Officer at the Bristol General Hospital. He then settled in practice at Knowle, where he had become a general favourite. He held at the time of his death the appointments of Honorary Anæsthetist to the Bristol General Hospital and Cossham Memorial Hospital and Medical Officer to the Bristol Speedway. His favourite sport was swimming, at which he had represented in turn the Universities of Cambridge and Bristol.

His early death is deeply deplored by all who knew him.

In 1937 he married Phyllis, daughter of Mr. Herbert Rogers, of Edgbaston.

